# The College of Nursing

**Published:** 1916-09-16

**Authors:** 


### THE MATRONS' AND SISTERS' DEPARTMENT.

### THE COLLEGE OF NURSING.

The Hon. Arthur Stanley is generously using the vacation in order to interest the nursing and hospital world up and down the country, and in Scotland, in the College of Nursing. He is really setting all the nursing world an example in energetic propaganda and self-denying devotion to the cause of trained and certificated nurses which ought to arouse every one of them to take action on their own behalf and speed up registration by sending in their names to Miss Rundle, at 6 Vere Street, London, W., and interesting their friends. Mr. Stanley is one of the busiest of men. He has had practically no holiday since the war began, and is now spending precious health-giving days in his brief holiday-time to help the nurses to help themselves. What are we to think of the quality and brain-power of the certificated trained nurses in these islands when we are told that they are considering whether they shall register their names because they do not want to pay a guinea without security—that is, until a Bill for the registration of trained nurses is passed? If this idea represents the average intelligence and capacity of the certificated trained nurse in Great Britain, Mr. Stanley's work may be thrown away. We believe, on the contrary, however, that British trained nurses are being steadily aroused, and that large numbers of them are keenly anxious for the establishment of the College of Nursing and the passing of the Registration Bill. That is why we venture to ask matrons and sisters to co-operate not passively, but actively, in helping Mr. Stanley in his self-denying efforts to help the whole nursing profession.

In some quarters it is suggested that, until the Registration Bill receives the Royal Assent, arrangements should be made for the nurses who wish to be on the register to complete their papers and send them in to the College of Nursing, 6 Vere Street, London, W., the date of payment of the fee being deferred until the date of the passing of the Act. There must be 30,000 nurses at least in Great Britain who are certificated, and at least half of them could certainly well afford to find a fee of one guinea if they were seriously set upon obtaining the right to put R.N. after their names. We are of opinion, therefore, that the registration fee for the first 15,000 nurses should be paid when their names are placed on the Register. We see, however, no objection to these fees being placed to a separate account and invested, pending the passing of the Registration Act within, say, twelve months or two years, the fee to be returnable to the nurse, without interest, failing the passing of the Act by a specified date. These questions are being talked about and discussed in nursing circles at the moment, and we think it best to bring them into the light of day and offer them for the public scrutiny of all sections of the nursing profession, including the matrons and sisters.

